# Neuronal correlates of attention and its disengagement in the superior colliculus of rat

**DOI:** 10.3389/fnint.2015.00009

**Published:** 2015-02-18

**Authors:** Nguyen H. Ngan, Jumpei Matsumoto, Yusaku Takamura, Anh H. Tran, Taketoshi Ono, Hisao Nishijo

**Affiliations:** System Emotional Science, Graduate School of Medicine and Pharmaceutical Sciences, University of ToyamaToyama, Japan

**Keywords:** attention, disengagement, single-unit recording, superior colliculus, rat

## Abstract

Orienting attention to a new target requires prior disengagement of attention from the current focus. Previous studies indicate that the superior colliculus (SC) plays an important role in attention. However, recordings of responses of SC neurons during attentional disengagement have not yet been reported. Here, we analyzed rat SC neuronal activity during performance of an attention-shift task with and without disengagement. In this task, conditioned stimuli (CSs; right and/or left light-flash or sound) were sequentially presented. To obtain an intracranial self-stimulation reward, rats were required to lick a spout when an infrequent conditioned stimulus appeared (reward trials). In the disengagement reward trials, configural stimuli consisting of an infrequent stimulus and frequent stimulus in the former trials were presented; in the non-disengagement reward trials, only an infrequent stimulus was presented. Of the 186 SC neurons responding to the CSs, 41 showed stronger responses to the CSs in the disengagement reward trials than in the non-disengagement reward trials (disengagement-related neurons). Furthermore, lick latencies in the disengagement reward trials were negatively correlated with response magnitudes to the CSs in half of the disengagement-related neurons. These disengagement-related neurons were located mainly in the deep layers of the SC. Another 70 SC neurons responded to the CSs in both disengagement and non-disengagement reward trials, suggesting that these neurons were involved in attention engagement. Our results suggest complementary mechanisms of attentional shift based on two subpopulations of neurons in the SC.

## INTRODUCTION

The superior colliculus (SC) constitutes part of the brain network involved in visual attention (Shipp, 2004; Krauzlis et al., 2013), and contributes to overt attention by controlling motor outputs (Sparks, 1999, 2002; Krauzlis, 2003) and target selection processes (McPeek and Keller, 2002, 2004; Nummela and Krauzlis, 2010). This structure also plays a crucial role in covert attention; inactivation of the primate SC impairs covert selection of signals for perceptual judgments (Lovejoy and Krauzlis, 2010). Further, microstimulation of the primate SC focuses attention without movement of eyes (Müller et al., 2005). Consistently, visuomotor neurons in the monkey SC were found active during covert shift of attention (Ignashchenkova et al., 2004). Non-invasive human imaging studies have also reported that the human SC is active during selective attention (Corbetta et al., 1991; Schneider and Kastner, 2009). These studies indicate a crucial role of the SC in the orientation of attention.

Orienting attention to a new target requires three sequential mental operations: (1) disengagement of attention from its current focus; (2) moving attention to the new target; and (3) engagement of the new target (Posner et al., 1984; Posner and Petersen, 1990). Thus, the process of attentional disengagement is a primary initial step in orienting. A behavioral study reported that reaction times to make a saccade to a peripheral target are faster when a central fixation point goes off shortly before target presentation (gap trials) than when the central fixation stimulus stays on (overlap trials; Saslow, 1967). This is because the subjects must disengage attention from the central target before shifting attention to the peripheral target in the overlap trials, and disengagement of attention takes time (Fischer and Breitmeyer, 1987). Despite the importance of the disengagement process, the neural mechanisms underlying disengagement processing are still poorly understood. To date, only four studies have focused on the neural mechanisms that are related to visual disengagement from fixation. These clinicopathological and electroencephalography (EEG) studies have suggested that the frontal eye fields (Rivaud et al., 1994) and parietal lobe (Posner et al., 1984; Csibra et al., 1997) might be involved in disengaging attention. Studying a patient with lesions in the right SC revealed that mean saccade latency to the contralateral peripheral target was longer than those in normal controls in overlap trials (Pierrot-Deseilligny et al., 1991). This suggests that the SC is also involved in attentional disengagement; the SC might be involved in “unlocking” of attention to a previous stimulus (e.g., a central fixation spot in overlap trials). However, no previous neurophysiological studies have investigated neural mechanisms of attentional disengagement in the SC. In the present study, we analyzed rat SC neuronal activity during performance of an attention-shift task with and without disengagement to acquire intracranial self-stimulation (ICSS) reward that mimic rewarding effects of natural foods (Ono et al., 1986). Here, we report that a population of SC neurons are involved in attentional disengagement processes.

## MATERIALS AND METHODS

### SUBJECTS

Eleven male Wistar rats, weighing 270–320 g at the time of surgery (12–16 weeks old; SLC, Hamamatsu, Japan), were used. The rats were individually housed in a room where temperature (24 ± 1^∘^C) and light (07:00–19:00) were automatically controlled. Food and water were available ad *libitum*. Treatment of all rats was in strict compliance with the United States Public Health Service Policy on Human Care and Use of Laboratory Animals, National Institutes of Health Guide for the Care and Use of Laboratory Animals, and Guidelines for the Care and Use of Laboratory Animals at the University of Toyama. All experimental procedures were approved by our institutional committee for experimental animal ethics.

### SURGERY

Surgery was performed under aseptic conditions in two stages. First, a cranioplastic cap was attached to the skull as described in our previous studies (Uwano et al., 1995; Nishijo et al., 1998). This cap was used for the head restraint system for wakeful rats and was identical to that of Nishijo and Norgren (1990). The rat was anesthetized (sodium pentobarbital, 40 mg/kg; intraperitoneal, i.p.,) and then mounted in a stereotaxic apparatus. The skull was exposed, and five small sterile stainless screws were threaded into holes in the skull to serve as anchors for cranioplastic acrylic. Two bipolar electrodes for ICSS were implanted in the peduncular part of the lateral hypothalamus (A, -3.36 from bregma; L, ±2.0; V, 9.2), according to the atlas of Paxinos and Watson (2007). Then, the cranioplastic acrylic was built up on the skull and molded around the conical ends of two sets of stainless steel bars. During subsequent surgery or during the recording session, the double end of these artificial earbars served the same function as regular earbars and could be used in the unanesthetized animal without inducing pain. A short length of 27-gage stainless-steel tubing was embedded into the cranioplastic acrylic near the bregma to serve as a reference pin. After surgery, an antibiotic (gentamicin sulfate, Gentacin®; injection, Schering-Plough, Osaka, Japan) was administered topically and systemically (2 mg; intramuscular, i.m.).

After recovery from surgery (5–7 day) and after training (7–10 days, see below), rats were again anesthetized (sodium pentobarbital, 40 mg/kg, i.p.) and mounted with the artificial earbars. A small hole (A, -8.0 to –5.0 from bregma; L, 0.0–2.0 right or left) was drilled through the cranioplastic acrylic and the underlying skull for chronic, repeated recordings. The exposed dura was excised, and the hole was covered with hydrocortisone ointment (Rinderon-VG®; ointment, Shionogi Co., Ltd., Tokyo, Japan); alternatively, one or two drops of chloramphenicol (Chloromycetin®; succinate, Sankyo Co., Ltd., Tokyo, Japan) solution (0.1 g/mL) were dropped into the hole. The hole was covered with a sterile Teflon sheet and sealed with epoxy glue.

### TRAINING AND TASK PARADIGMS

Before surgery, the rats were acclimated by handling and were accustomed to being placed into a small, plastic restraining cage for brief periods. After recovery from the first stage of surgery, the threshold level for ICSS (0.5 s train of 100 Hz, 0.3 ms capacitor-coupled negative square-wave pulses) was determined, and any rat for which the threshold exceeded 300 μA was excluded. In these ICSS parameters, rats produced stable 40–70 lever presses/min for ICSS in an operant chamber (Kobayashi et al., 2003). Then, the rat was trained to perform the attention-shift task with and without attentional disengagement.

**FIGURE 1:**
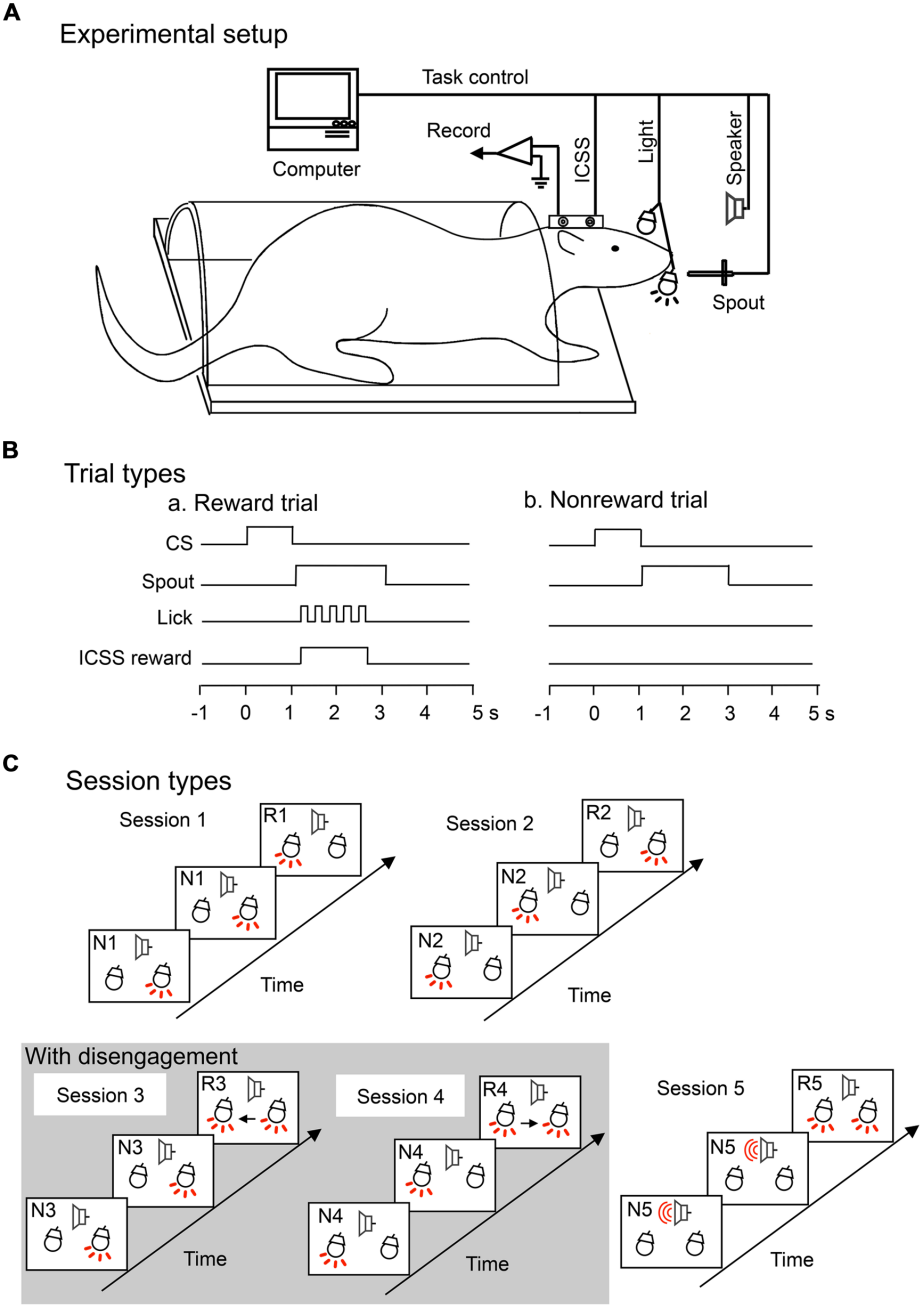
**Experimental setup **(A)** and trial **(B)** and session **(C)** types for an attention-shift task. (A)** Experimental setup. Rats were prepared for chronic recording by forming receptacles of dental cement to accept artificial earbars. Electrodes were implanted in the lateral hypothalamic area for intracranial self-stimulation (ICSS) of the medial forebrain bundle. The rat was trained to lick when the spout was automatically placed close to its mouth. Auditory and visual conditioned stimuli (CSs) were presented by a speaker in front of its head and a light in front of each eye, respectively. **(B)** Trial types. In each trial, the CS appeared for 1 s, followed by spout protrusion close to the mouth for 2 s. In the reward (a) but not non-reward (b) trials, rats could obtain an ICSS reward if the rats licked the spout. **(C)** Session types. The task included five sessions. In session 1, right light (frequent stimulus) was sequentially presented, and when the left light, an infrequent stimulus, appeared, the rat could acquire reward if it licked the spout. In session 2, left light (frequent stimulus) was sequentially presented, and when the right light (infrequent stimulus) appeared, the rat could acquire reward if it licked the spout. In sessions 3 and 4, right and left lights (frequent stimuli) were similarly presented, respectively. However, the infrequent stimuli appeared with the frequent stimuli. Therefore, in sessions 3 and 4, the rat must disengage attention from the frequent stimulus. Arrows show attention disengagement and subsequent direction of attentional shift. Lights and speaker with the red-colored lines indicate that these apparatuses are turned on. In session 5, tone (frequent stimulus) was sequentially presented, and when both right and left lights (infrequent stimuli) were simultaneously presented, the rat could acquire reward if it licked the spout. Note that the infrequent stimuli in session 5 were the same as those in sessions 3 and 4, but attentional disengagement was not required in session 5. R1–R5, reward trials in sessions 1–5; N1–N5, non-reward trials in sessions 1–5.

During task training, the rat was placed in the restraining cage with its head fixed rigidly and painlessly in the stereotaxic device by the artificial ear bars. A midrange speaker, located 1 m in front of the rat, delivered the auditory stimuli, and each white light, 3 cm in front of each eye, delivered the visual stimuli (**Figure 1A**). The attention-shift task included five sessions, and each session consisted of 36 trials including 12 reward trials (infrequent trials) and 24 non-reward trials (frequent trials). In each trial, a CS (light flash or sound) appeared for 1 s, followed by spout protrusion close to the mouth for 2 s. In the reward trials (but not non-reward trials), rats could obtain ICSS reward if they licked the spout (**Figure 1B**). A touch sensor detected individual spout licks.

The sequence of the CSs in each session are shown in **Figure 1C**. In all sessions, two kinds of CSs (infrequent and frequent) were sequentially presented, and when rats detected infrequent CSs and licked the spout, rats could acquire ICSS reward (reward trials). Trial sequence was set sub-randomly by a computer in that at least one non-reward trial always preceded each reward trial. Thus, the task required shift of attention to infrequent CSs. In session 1, right light associated with non-reward was sequentially presented (non-reward trials), and when the left light, an infrequent stimulus, appeared, the rat could acquire ICSS reward if it licked the spout (reward trials). In session 2, left light associated with non-reward was sequentially presented (non-reward trials), and when right light, an infrequent stimulus, appeared, the rat could acquire ICSS reward if it licked the spout (reward trials). In sessions 3 and 4, right, or left light associated with non-reward was similarly sequentially presented (non-reward trials), and when both right and left lights were simultaneously presented, the rat could acquire ICSS reward if it licked the spout (reward trials). The CS in the reward trials included not only the infrequent stimuli but also frequent stimuli of the former non-reward trials. Therefore, in sessions 3 and 4, the rat must disengage attention from the frequent CSs to detect the infrequent stimuli. In session 5, a tone (frequent stimulus) was sequentially presented, and when both right and left lights (infrequent stimuli) were simultaneously presented, the rat could acquire reward if it licked the spout. Session 5 was used as a control session for sessions 3 and 4; the CSs in the reward trials in session 5 were same as those in sessions 3 and 4, although attentional disengagement from the CSs in the former trials was not required in session 5. SC neurons related to attention disengagement are supposed to respond stronger to the CSs in sessions 3 and/or 4 than in session 5 (e.g., **Figure 3** for attention disengagement-related neurons). Sequence of the sessions was run pseudo-randomly. The rats were trained to lick the spout only in the reward trials.

### ELECTROPHYSIOLOGICAL PROCEDURES AND DATA ACQUISITION

After the rats had learned the conditioned licking task to discriminate the CSs in the reward and non-reward trials, SC neurons were recorded during performance of the task. Neuronal activity of an individual rat was usually recorded every other day. After being placed in the enclosure, the ointment was removed, and a glass-insulated tungsten microelectrode (*Z* = 0.5–1.5 MΩat 1 kHz) was stereotaxically and vertically inserted into the SC in a stepwise fashion by a pulse motor-driven manipulator (SM-20, Narishige, Tokyo, Japan). Extracellular neuronal activity was passed through a multi-channel differential amplifier with a preamplifier (PBX, Plexon Inc., Dallas, TX, USA), monitored on an oscilloscope, and recorded on a data recorder (RD-135T DAT DATA RECORDER, TEAC). Only neuronal activity with a signal-to-noise ratio >3:1 was recorded. The analog signals of amplified neuronal activity, triggers for CSs, ICSS reward, and spout licking were digitized at a 40-kHz sampling rate and stored on a computer via a multichannel acquisition processor system (MAP, Plexon Inc.). The digitized neuronal activities were isolated into single units by their waveform components using the Oﬄine Sorter program (Plexon). Spike sorting was performed with the oﬄine sorter program for cluster analysis (Oﬄine Sorter). Each cluster was checked manually to ensure that the cluster boundaries were well separated and the waveform shapes were consistent with action potentials. For each isolated cluster, an autocorrelogram was constructed and only units with refractory periods >1.2 ms were used for further analyses. Finally, superimposed waveforms of the isolated units were drawn to check the consistency of the waveforms. These units were transferred to the NeuroExplorer program (Nex Technologies, Littleton, MA, USA) for further analyses.

### ANALYSIS OF THE BASIC CHARACTERISTICS OF SC NEURONS

Since the rats had to adapt to new rules in the beginning of the sessions, initial trials in each session were discarded and only data after the third reward trial were analyzed. Both neuronal and behavioral data on each trial were counted from the peristimulus histograms in successive 50-ms bins for three periods: a pretrial control period (500 ms), a CS stimulation period (1,000 ms), and rewarding stimulation (reinforcement) period (2,000 ms). Significant excitatory or inhibitory responses to each CS were defined by a Wilcoxon signed rank (WSR) test (*P* < 0.05) of neuronal activity between the 500-ms pretrial control period and the 1,000-ms CS periods. Neuronal response magnitude was defined as follows: the mean firing rate during the 1,000-ms CS period minus the mean firing rate during the 500-ms pretrial period. For each neuron, neuronal response magnitudes to all CSs were compared by the one-way analysis of variance (ANOVA) and Tukey *post hoc* tests (*P* < 0.05). Based on response patterns to the CSs, we then classified SC responsive neurons (see Results).

To analyze latencies of neuronal responses, one peri-event histogram (in 10-ms bins) was constructed using the data of the whole reward and non-reward trials across five sessions in each neuron. Neuronal response latency was defined as the interval from the onset of stimulus presentation to the time at which neuronal firing rate exceeded the mean ± 2.0 SD of the baseline firing rate. Mean response latencies to the CSs were compared among SC neuronal types by one-way ANOVAs at a significance level of *P* < 0.05. The *post hoc* comparisons were performed using Tukey tests with a significance level of *P* < 0.05. All data are expressed as mean ± SEM.

For some SC neurons (attentional disengagement-related neurons, see Results), not only response magnitudes, but also response latencies and durations were analyzed in each CS. For this purpose, peri-event histograms of individual CSs were constructed. Neuronal response duration was defined as the duration during which the neuronal firing rate exceeded the mean ± 2.0 SD of the baseline firing rate. Because these neurons showed no responses in the non-reward trials, we analyzed latencies and durations of neuronal responses to each stimulus only in the reward trials. Mean latencies and duration of responses to the CSs were similarly compared among the CSs by one-way repeated-measure ANOVAs at a significance level of *P* < 0.05.

### ANALYSIS OF LICK LATENCIES AND THEIR CORRELATION WITH NEURONAL ACTIVITY

Lick latency was defined as the interval from spout protrusion to the moment when the rat licked the spout. Mean lick latencies in reward trials were compared among the five sessions by one-way repeated-measure ANOVAs. The *post hoc* comparisons were performed using the Bonferroni-correction method with a significance level of *P* < 0.05. Correlations between individual lick latencies in the reward trials and neuronal activity were analyzed using simple linear regression.

### HISTOLOGY OF THE RECORDING SITES

After the last recording session, rats were anesthetized with pentobarbital (50 mg/kg, i.p.), and several small electrolytic lesions (20 μA for 20 s) were made stereotaxically around the recording sites with a glass-insulated tungsten microelectrode. Rats were then given an additional overdose of anesthetic and perfused transcardially with heparinized 0.9% saline followed by 10% buffered formalin. The brain was removed and cut into 50-μm frontal sections with a freezing microtome. Sections were Nissl-stained with cresyl violet. The sites of electrical lesions were carefully determined microscopically. The location of each recording site was then calculated by comparing the stereotaxic coordinates of the recording sites with those of the lesions. Positions of neurons were stereotaxically located on the tissue sections and plotted on the corresponding sections on the atlas of Paxinos and Watson (2007).

Based on the locations of SC neurons, the ratio of SC neurons in the superficial layers, the intermediate layers and the deep layers were calculated for each type of SC neuron. The ratio (percent) of each layer for each type of SC neuron was defined as follows: (the number of a given type of SC neuron × 100)/the number of SC neurons recorded in that layer. The ratio of SC neurons among the different SC layers was compared with a Chi-squared test (*P* < 0.05).

## RESULTS

### BEHAVIORAL RESULTS

**FIGURE 2:**
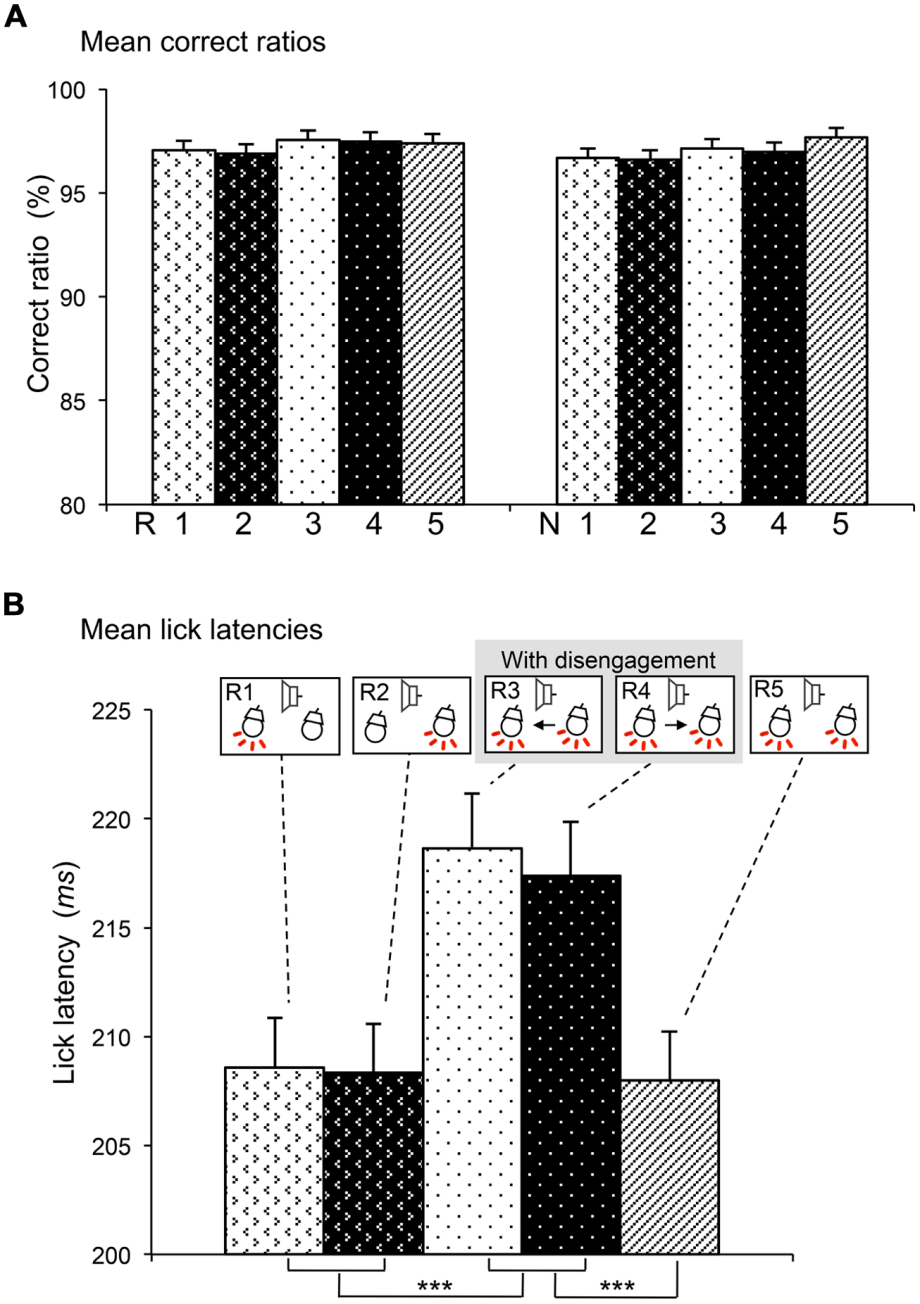
**Comparisons of correct ratios in the reward and non-reward trials **(A)** and lick latencies in the reward trials **(B)** among the five sessions. (A)** There was no significant differences in correct ratios (two-way ANOVA, *P* > 0.05). R1–5, reward trials in sessions 1–5; N1–5, non-reward trials in sessions 1–5. **(B)** Lick latencies were significantly longer in sessions 3 and 4 than other sessions. ***significant difference from sessions 1, 2, and 5 (Bonferroni test after one-way repeated measures ANOVA, *P* < 0.001). The behavioral data were collected during the recordings of 156 neurons.

During the task, rats almost always licked the spout in the reward trials, while they seldom licked the spout in the non-reward trials. We analyzed the behavioral data that were collected during recordings of 156 responsive SC neurons. **Figure 2A** shows the mean correct ratios in the reward and non-reward trials of the five sessions. Mean correct ratios were high (97.27 and 97.02% in the reward and non-reward trials, respectively). Statistical comparison indicated that there was no significant main effect of session [*F*(4,1550) = 1.050, *P* > 0.05], nor significant interaction between session and trial type (reward or non-reward trial; *F*(4,1550) = 0.221, *P* > 0.05; two-way ANOVA). The results indicated that the rats similarly performed the five sessions.

Lick latencies were analyzed in the reward trials. We analyzed the behavioral data with lick latencies less than 300 ms, which were collected during recordings of 156 responsive SC neurons. **Figure 2B** shows the mean lick latencies in the reward trials of the five sessions (R1–R5). Statistical comparison indicated that there was a significant main effect of session [*F*(4,152) = 23.216, *P* < 0.001] (one-way repeated-measures ANOVA). *Post hoc* tests indicated that mean lick latencies in sessions 3 and 4 requiring disengagement (R3, R4) were significantly longer than those in the other sessions requiring no disengagement (R1, R2, and R3; Bonferroni tests, *P* < 0.001).

### CLASSIFICATION OF SC NEURONS

Over a period of 1–3 months for each rat, recordings were made from 611 neurons located in and around the SC during the attention-shift task. Of these neurons, 583 were located in the SC. **Table 1** summarizes the response patterns of these 583 neurons. One hundred and eighty-six (31.9%) neurons responded to the CSs. These 186 responsive neurons were classified into four types: disengagement-related neurons (22.0%, 41/186), reward and attention shift-related neurons (37.6%, 70/186), visually responsive neurons (33.9%, 63/186), and inhibitory-responsive neurons (6.5%, 12/186).

**Table 1:** Classification and numbers of the SC neurons.

Classification	Number of neurons (R/L)			
	Superficial layers	Intermediate layers	Deep layers	Total
Disengagement-related	2 (1/1)	10 (5/5)	29 (13/16)	41 (19/22)
Reward and attention shift-related	7 (3/4)	29 (15/14)	34 (16/18)	70 (34/36)
Visually responsive	40 (18/22)	18 (9/9)	5 (3/2)	63 (30/33)
Inhibitory responsive	5 (3/2)	4 (2/2)	3 (2/1)	12 (7/5)
Total responses	54 (25/29)	61 (31/30)	71 (34/37)	186 (90/96)
No responses	158 (76/82)	120 (57/63)	119 (57/62)	397 (190/207)
Total	212 (101/111)	181 (88/93)	190 (91/99)	583 (280/303)

R, right superior colliculus; L, left superior colliculus. Numbers in parentheses indicate numbers of neurons in the right (R) and left (L) sides.

### ATTENTION DISENGAGEMENT-RELATED NEURONS

**FIGURE 3:**
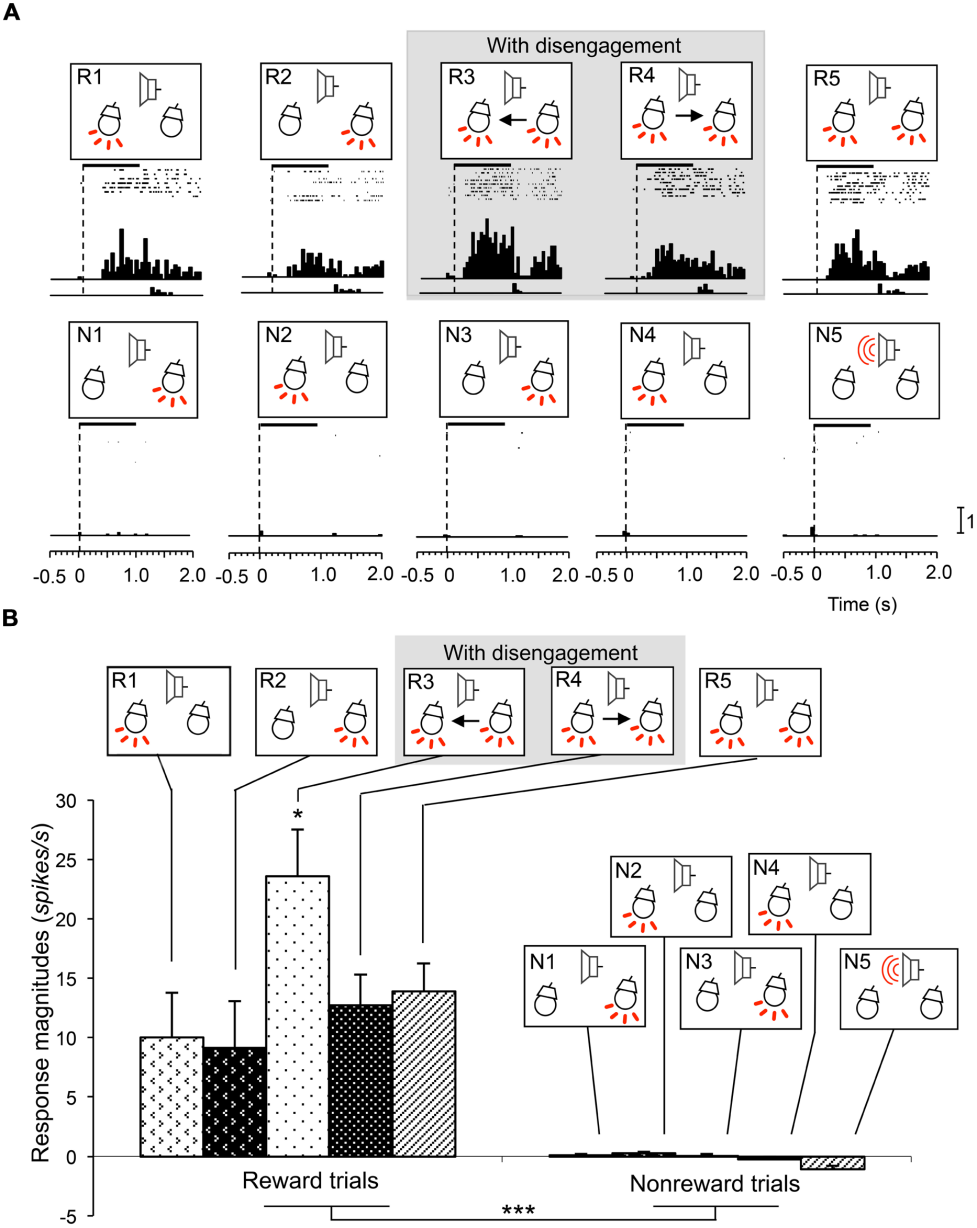
**An example of an attention disengagement-related neuron recorded from the right SC. (A)** Raster displays of neuronal activity and summed histograms in response to each stimulus. R1–R5 represent neuronal responses to the infrequent CSs associated with reward, and N1–N5 represent neuronal responses to the frequent CSs associated with non-reward. Horizontal bars above the raster displays indicate the stimulus presentation periods (1.0 s). Vertical dotted line in each of the raster displays and histograms indicates stimulus onset. Calibration at the right bottom of the figure indicates the number of spikes per trial in each bin. Bin width, 50 ms. **(B)** Comparison of response magnitudes of the neuron shown in A to the CSs. This neuron responded stronger to the CSs in session 3 (R3). *significant difference from the CSs in R1, R2, R4, and R5 (Tukey test, *P* < 0.05); ***significant difference from the CSs in N1–N5 (Tukey test, *P* < 0.001).

Attention disengagement-related neurons were defined as such if the neurons satisfied the following two comparisons; if neurons showed excitatory responses during presentation of the infrequent CSs requiring attentional disengagement (CSs in the reward trials in sessions 3 and 4) contralateral to the recording sites (WSR test, *P* < 0.05), and if they also showed significantly higher response magnitudes during presentation of these CSs than other CSs associated with and without reward (Tukey test after one-way ANOVA, *P* < 0.05). A typical example of this type of neuron in the right SC is shown in **Figure 3A**. The neuron responded during presentation of all infrequent CSs associated with reward in sessions 1–5 (**Figure 3A**, R1–R5; WSR test, *P* < 0.05), but not during presentation of frequent CSs associated with non-reward (**Figure 3A**, N1–N5; WSR test, *P* > 0.05). Comparisons of the response magnitudes to the CSs are shown in **Figure 3B**. The response magnitudes during presentation of the infrequent CSs contralateral to the recording site requiring attention disengagement in session 3 (R3) were significantly stronger than those to the other CSs with and without disengagement (Tukey test after one-way ANOVA, *P* < 0.01). It is noted that this SC neuron was recorded from the right SC, and the left light was the infrequent CS in session 3. To detect the left light associated with reward, the rat must disengage attention from the right light in session 3. On the other hand, the rat had to detect the left light in session 1 and the left and right lights in session 5, which required no disengagement. The above results indicate that the response magnitude to the CS requiring disengagement in session 3 was significantly larger than to the same CSs requiring no disengagement in sessions 1 and 5.

**FIGURE 4:**
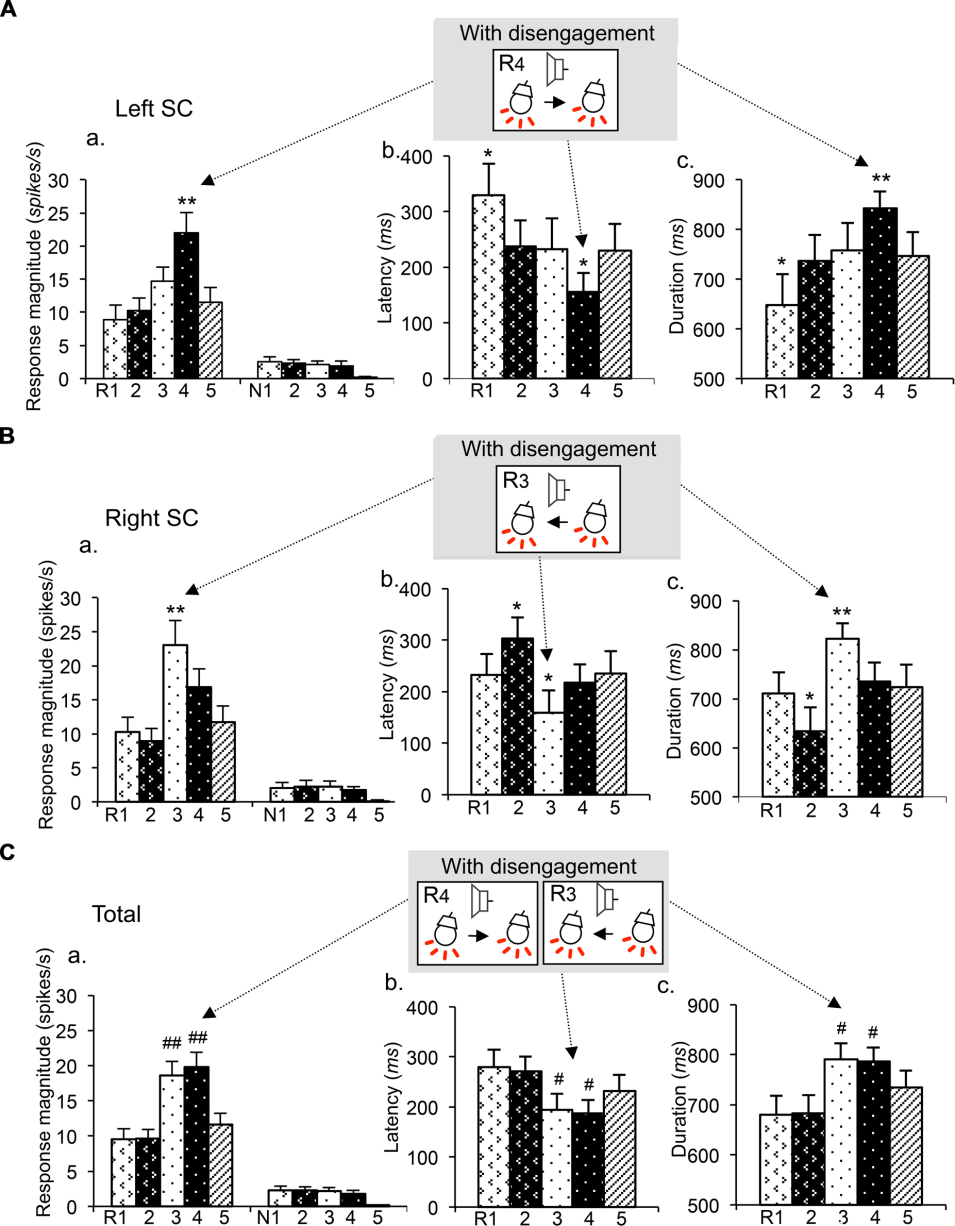
**Mean response magnitudes (a), latencies (b), and durations (c) of the attention disengagement-related neurons. (A)** Attention disengagement-related neurons (*n* = 22) recorded from the left SC. The mean response magnitude to the CSs was stronger (a), the mean response latency was shorter (b), and the mean response duration was longer (c) in session 4 than other sessions. **, *significant differences from the CSs in the reward trials of other sessions (Bonferroni test, *P* < 0.01, *P* < 0.05, respectively). **(B)** Attention disengagement-related neurons (*n* = 19) recorded from the right SC. The mean response magnitude to the CSs was stronger (a), the mean response latency was shorter (b), and the mean response duration was longer (c) in session 3 than other sessions. **, *significant differences from the CSs in the reward trials of other sessions (Bonferroni test, *P* < 0.01, *P* < 0.05, respectively). **(C)** Attention disengagement-related neurons (*n* = 41) recorded from both sides of the SC. The mean response magnitude to the CSs was stronger (a), the mean response latency was shorter (b), and the mean response duration was longer (c) in sessions 3 and 4 than other sessions. ##, # significant differences from the CSs in the reward trials of sessions 1, 2, and 5 (Bonferroni test, *P* < 0.01, *P* < 0.05, respectively).

**Figure 4** shows mean response magnitudes (a), mean response latencies (b), and the mean response durations (c) of the disengagement-related neurons recorded from the left SC (A), right SC (B), and both sides of the SC (C). In the left SC neurons (*n* = 22; A), the mean response magnitude was significantly stronger (a), the mean response latency was significantly shorter (b) and the mean response duration was significantly longer in session 3 requiring disengagement than those in other sessions (Bonferroni test after one-way repeated-measures ANOVA; *P* < 0.01, *P* < 0.05, and *P* < 0.01, respectively). In the right SC neurons (*n* = 19; B), the mean response magnitude was significantly stronger (a), the mean response latency was significantly shorter (b) and the mean response duration was significantly longer in session 4 requiring disengagement than those in other sessions (Bonferroni test after one-way repeated-measures ANOVA; *P* < 0.01, *P* < 0.05, and *P* < 0.01, respectively). In SC neurons bilaterally (*n* = 41) (C), the mean response magnitude was significantly stronger (a), the mean response latency was significantly shorter (b), and the mean response duration was significantly longer in sessions 3 and 4 requiring disengagement than those in other sessions (Bonferroni test after one-way repeated-measures ANOVA; *P* < 0.01, *P* < 0.05, and *P* < 0.05, respectively). These results indicate that the disengagement-related neurons responded stronger and faster to the CSs in the contralateral visual field requiring attentional disengagement.

**FIGURE 5:**
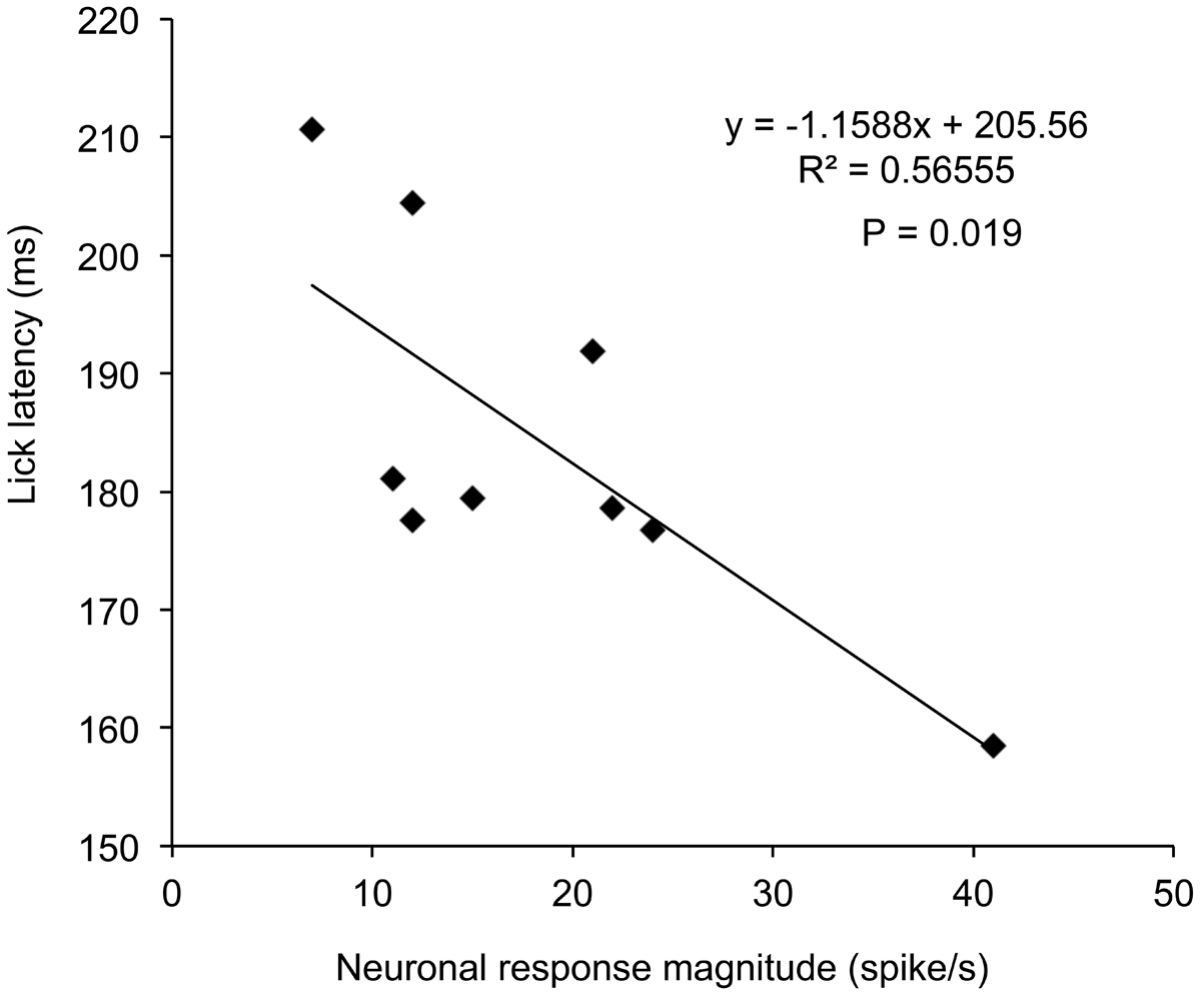
**Relationships between response magnitudes to the CSs and lick latencies in session 3 in the disengagement-related neuron shown in **Figure 3**.** There was a significant negative correlation between the response magnitudes and lick latencies (*P* = 0.019, simple linear regression).

The above results suggest that activity of this type neurons might correlate with lick behaviors in the sessions requiring disengagement. **Figure 5** shows the relationships between neuronal response magnitudes in R3 shown in **Figure 3** and lick latencies in individual trials. Statistical analysis by simple linear regression indicated that there was a significant negative correlation between response magnitudes and lick latencies [(*F*(1,7) = 9.11, *P* = 0.019; *r* = –0.75)]. Thus, stronger neuronal responses were accompanied by the shorter licking latencies. Of the 41 disengagement-related neurons, 17 (17/41, 41.5%) [left SC, 9/22 (40.9%); right SC, 8/19 (42.1%)] showed similar significant negative correlations between neuronal response magnitudes in session 3 or 4 with disengagement and lick latencies (*r* ranging from –0.68 to –0.93; *P* < 0.05, simple linear regression). Furthermore, another five neurons tended to show similar significant negative correlations (*r* ranged from –0.60 to –0.78; *P* < 0.1, simple linear regression). These results indicate that the attention disengagement-related neurons guide behaviors in the trials requiring attentional disengagement.

### REWARD AND ATTENTION SHIFT-RELATED NEURONS

**FIGURE 6:**
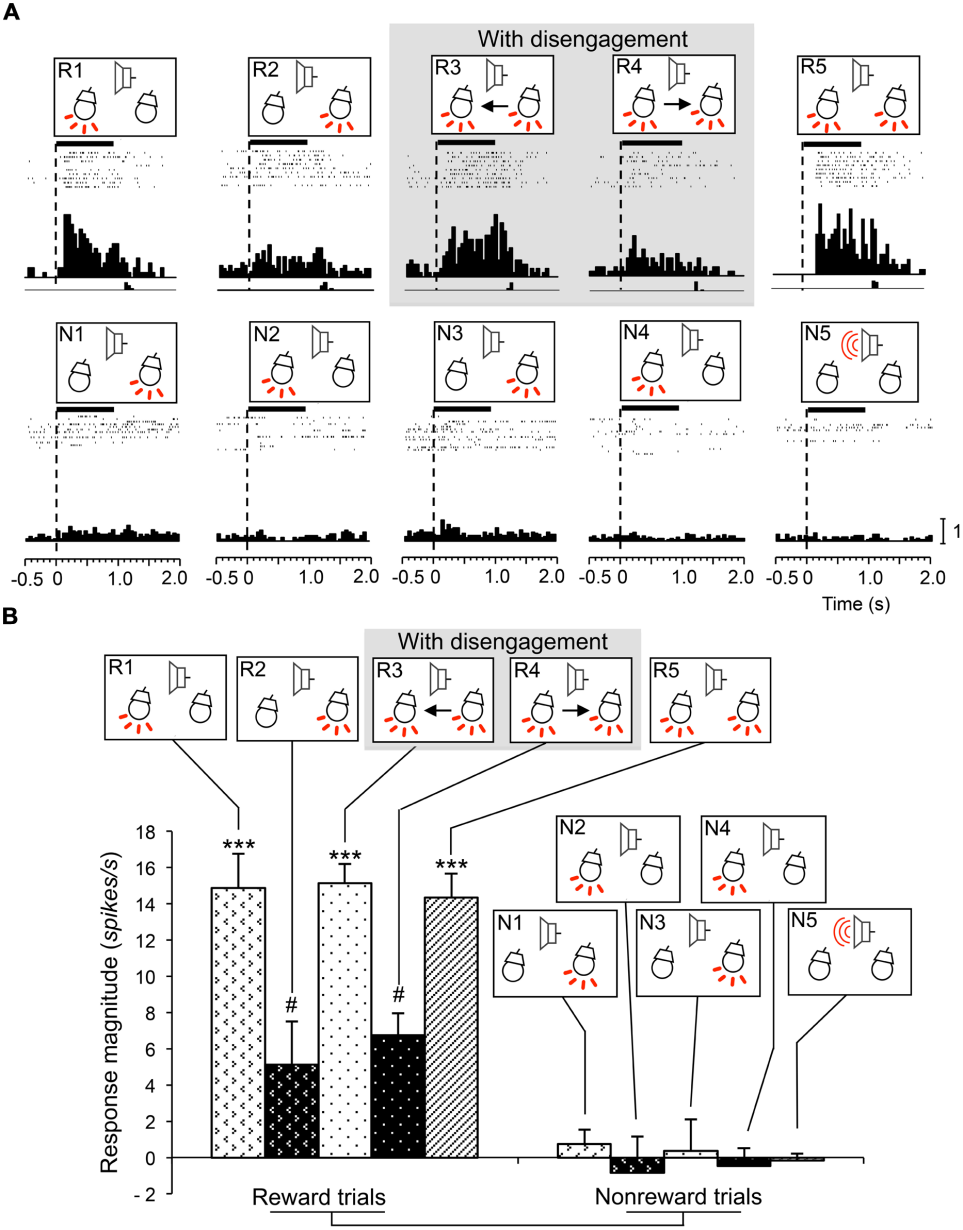
**An example of a reward and attention shift-related neuron recorded from the right SC. (A)** Raster displays of neuronal activity and summed histograms in response to each stimulus. The neuron responded to the infrequent CSs in the reward trials, but not to the frequent CSs in the non-reward trials. **(B)** Comparison of response magnitudes of the neuron shown in A to the CSs. ***significantly different from the CSs the CSs in N1 to N5 (Tukey test, *P* < 0.001); # significantly different from the CSs in R1, R3, and R5 (Tukey test, *P* < 0.05). Other descriptions are the same as for **Figure 3**.

Reward and attention shift-related neurons were defined as neurons that showed excitatory responses during presentation of the infrequent CSs in reward trials contralateral to the recording sites (e.g., CSs in R1, R2, and R3 for the SC neurons in the right SC; WSR test, *P* < 0.05), regardless of attentional disengagement, and if response magnitudes to these CSs were larger than those to the frequent CSs without reward (Tukey test after one-way ANOVA, *P* < 0.05). In 46 of the 70 reward and attention shift-related neurons, responses to the infrequent CSs contralateral to the recording sites were larger than those to the infrequent CSs ipsilateral to the recording sites (Tukey test after one-way ANOVA, *P* < 0.05). A typical example of this type of neuron recorded from the right SC is shown in **Figure 6**. This neuron showed excitatory responses during presentation of all infrequent CSs including those contralateral to the recording site (CSs in R1, R3, and R5) that were associated with reward (WSR test, *P* < 0.01), and not to the frequent CSs associated with non-reward (WSR test, *P* > 0.05) (A). Comparison of the response magnitudes to the CSs are shown in **Figure 6B**. The response magnitudes to the infrequent CSs contralateral to the recording site (CSs in R1, R3, and R5) were significantly stronger than that to the CSs associated with non-reward CSs (Tukey test, *P* < 0.001), and also larger than those to the infrequent CSs ipsilateral to the recording site (CSs in R2 and R4; Tukey test, *P* < 0.05). We found that the response magnitudes to the configural CSs in session 4, in which left light was the frequent stimuli (i.e., non-target), was significantly smaller than those to the same configural CSs in session 3, in which left light was the infrequent stimuli (i.e., target). These results indicate that this neuron responded stronger to the same contralateral CS (left light) when the rat attended to it than when the rat did not attend to it, suggesting that activity of this neuron reflects visual attention.

### VISUALLY RESPONSIVE NEURONS

**FIGURE 7:**
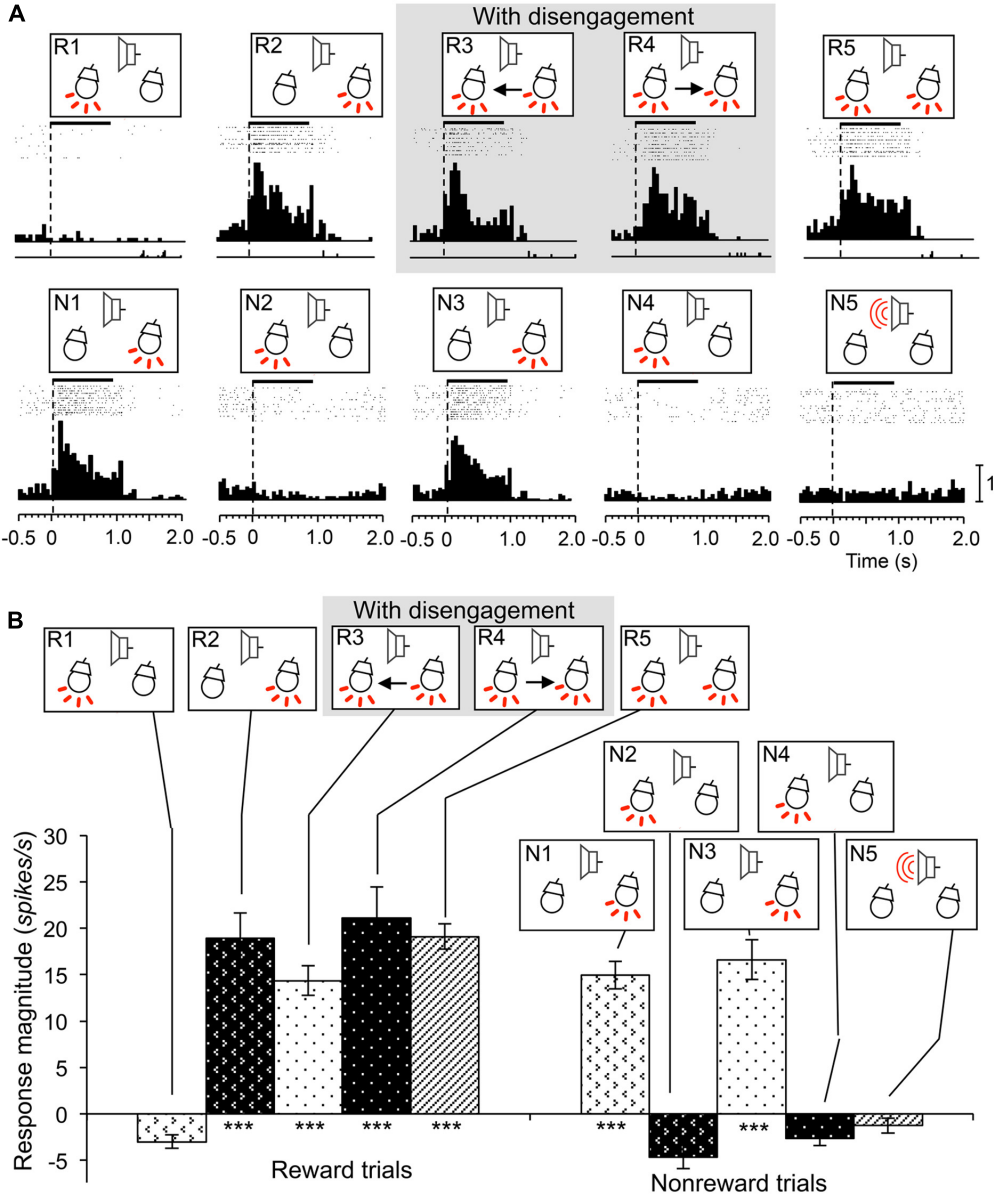
**An example of a visually responsive neuron recorded from the left SC. (A)** Raster displays of neuronal activity and summed histograms in response to each stimulus. The neuron responded to the all CSs that included right light regardless of the session and reward association. **(B)** Comparison of response magnitudes of the neuron shown in A to the CSs. ***significant difference from the CSs that did not include the right light (Tukey test, *P* < 0.001). Other descriptions are the same as for **Figure 3**.

Visually responsive neurons were defined as neurons that showed excitatory responses (WSR test, *P* < 0.05) to the visual CSs (light flash) contralateral to the recording sites regardless of reward association. A typical example of this type of neuron recorded from the left SC is shown in **Figure 7**. The neuron responded to the CSs that included the right light (R2-5, N1, and N3) regardless of reward association (WSR test, *P* < 0.001; A). Comparison of the response magnitudes to the CSs are shown in **Figure 7B**. The response magnitudes to the CSs that included the right light were significantly stronger compared to those to the other CSs (Tukey test after one-way ANOVA, *P* < 0.001).

### INHIBITORY-RESPONSIVE NEURONS

**FIGURE 8:**
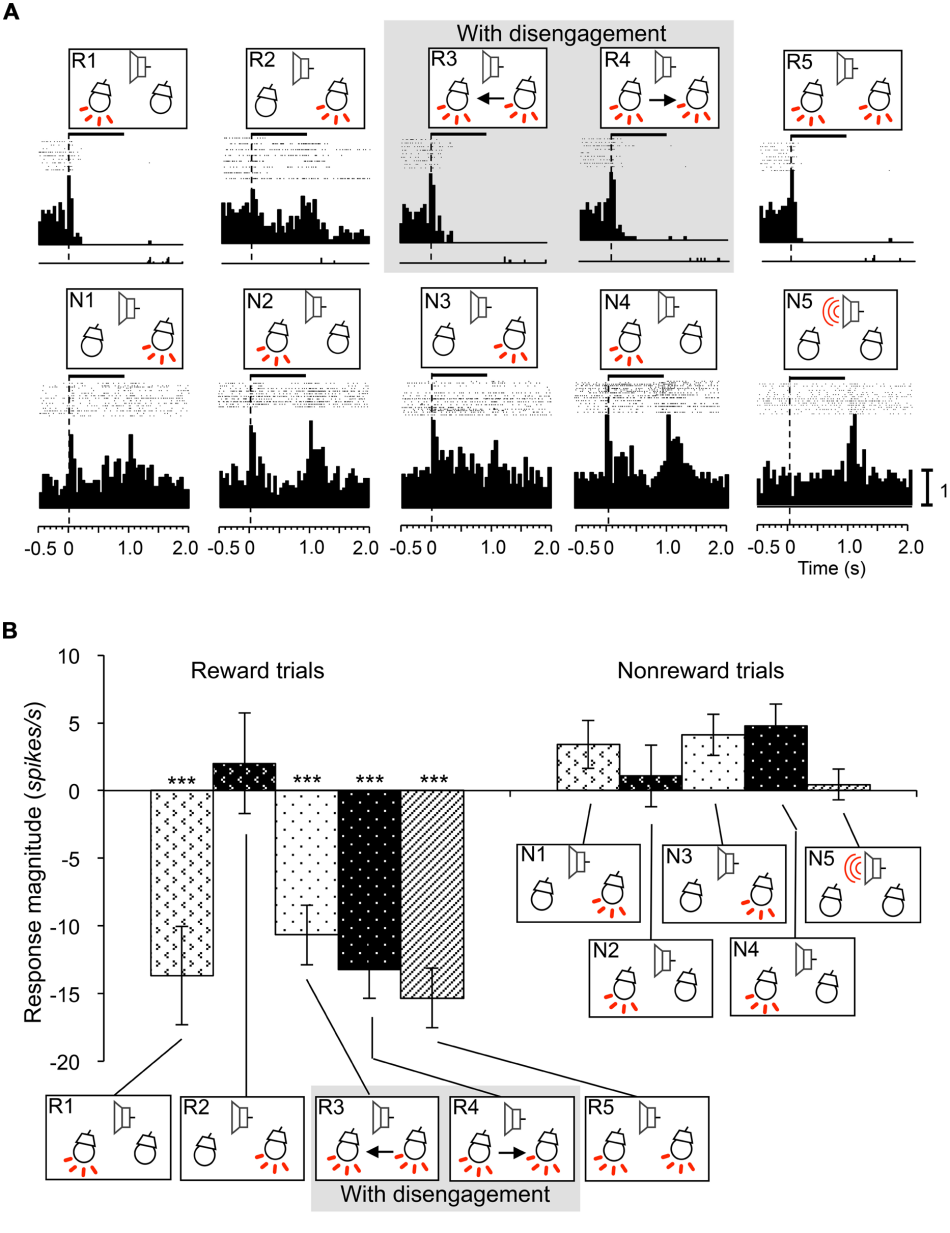
**An example of an inhibitory responsive neuron recorded from the right SC. (A)** Raster displays of neuronal activity and summed histograms in response to each stimulus. The neuron showed inhibitory responses to the all CSs that included left light in the reward trials. **(B)** Comparison of response magnitudes of the neuron shown in A to the CSs. ***significant difference compared to the CSs in the reward trials of session 2 and all the CSs in the non-reward trials (Tukey test, *P* < 0.001). Other descriptions are the same as for **Figure 3**.

Inhibitory-responsive neurons were defined as neurons that showed inhibitory responses to some of the CSs (WSR test, *P* < 0.05). This type of SC neuron sometimes showed transient excitatory responses to those CSs in short latencies. Of the 12 inhibitory-responsive neurons, four neurons showed inhibitory responses to the CSs associated with reward if the CSs included the light in the contralateral visual field. These neurons showed responses similar to reward and attention shift-related neurons. A typical example of this type neuron recorded from the right SC is shown in **Figure 8**. This neuron showed inhibitory responses to the infrequent CSs contralateral to the recording site that were associated with reward regardless of attention disengagement (WSR test, *P* < 0.001), and not to the frequent CSs associated with non-reward (WSR test, *P* > 0.05; A). Comparison of the response magnitudes to the CSs are shown in **Figure 8B**. The absolute values of the response magnitudes to the infrequent CSs contralateral to the recording site were significantly stronger than responses to the other CSs (Tukey test after one-way ANOVA, *P* < 0.001). The remaining eight inhibitory-responsive neurons showed inhibitory responses to the contralateral light regardless of reward.

### RESPONSE LATENCIES AND RECORDING SITES OF SC NEURONS

The mean response latency of the visually responsive neurons was short (26.5 ± 0.7 ms). However, attention disengagement-related neurons (157.2 ± 21.9 ms), reward and attention shift-related neurons (130.4 ± 13.3 ms), and inhibitory-responsive neurons (123.0 ± 20.2 ms) showed significantly longer mean response latencies (Tukey test after one-way AVOVA, *P* < 0.001).

**FIGURE 9:**
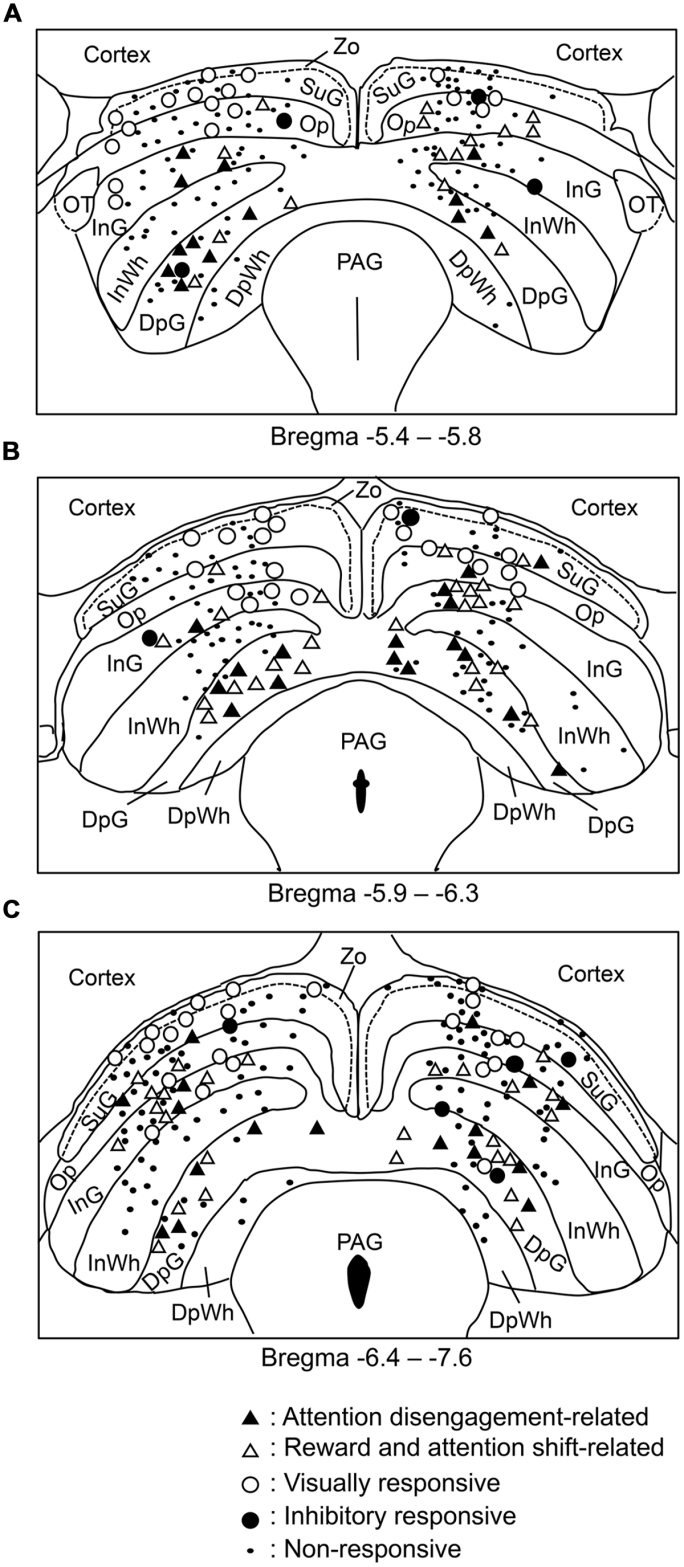
**Distributions of all neurons recorded from the SC. (A–C)** Coronal sections, based on the atlas of Paxinos and Watson (2007). Values below each section indicate distance (mm) from the bregma. DpG, deep gray layer; DpWh, deep white layer; InG, intermediate gray layer; InWh, intermediate white layer; Op, optic nerve layer; PAG, periaqueductal gray; SuG, superficial gray SC; Zo, zonal layer.

**FIGURE 10:**
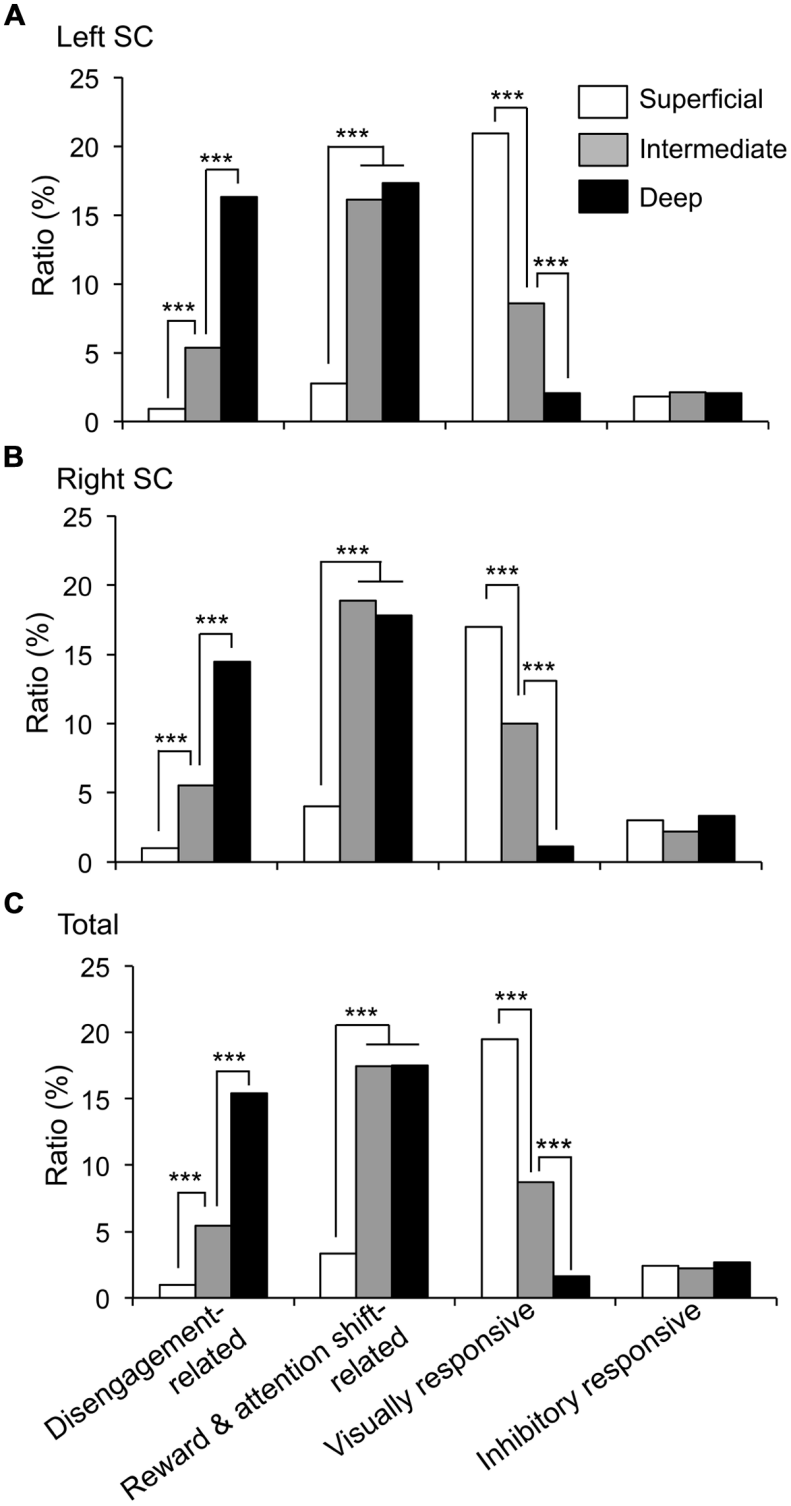
**Ratios of each neuronal type in the superficial, intermediate, and deep layers of the SC. (A–C)** Ratios of the neurons in each layer in the left SC **(A)**, right SC **(B)** and both sides of the SC **(C)**. ***significant difference (Chi-squared test, *P* < 0.001).

The recording sites of all SC neurons are shown in **Figure 9**. Of the 583 SC neurons recorded, 212 were located in the superficial layers (Zo, SuG, Op), 181 in the intermediate layers (InG, InWh), and 190 in the deep layers (DpG, DpWh). The ratios of neurons in each layer of each neuronal type are shown in **Figure 10**. For the disengagement related-neurons, the ratios of the deep layers were significantly higher than those of the intermediate and the superficial layers (Chi-squared test, *P* < 0.001), and the ratios of the intermediate layers were significantly higher than those of the superficial layers (Chi-squared test, *P* < 0.001) in the left SC (A), right SC (B), and both sides of the SC (C). These results indicate that the disengagement-related neurons were located mainly in the deep layers. For the reward and attention shift-related neurons, the ratios of the deep layers as well as the intermediate layers were significantly higher than those of the superficial layers (Chi-squared test, *P* < 0.001) in the left SC (A), right SC (B), and both sides of the SC (C). There were no significant differences in the ratios between the deep and intermediate layers (Chi-squared test, *P* > 0.05). These results indicate that the reward and attention shift-related neurons were located mainly in both the deep and intermediate layers. For the visually responsive neurons, the ratios of the superficial layers were significantly higher compared to the intermediate layers (Chi-squared test, *P* < 0.001), and the ratios of the intermediate layers were significantly higher compared to that of the deep layers (Chi-squared test, *P* < 0.001). Visually responsive neurons were mainly located in the superficial layers.

## DISCUSSION

### VALIDITY OF THE BEHAVIORAL PARADIGM

In the present study, to induce attention to a specific side, the frequent stimulus was repeatedly presented to that given side in sessions 1–4. Then, the infrequent stimulus was presented in the opposite side simultaneously with the frequent stimulus in sessions 3 and 4, while the infrequent stimulus was presented alone in the opposite side in sessions 1 and 2. Therefore, to engage attention to the infrequent stimulus in the opposite target side in sessions 3 and 4, the animals were required to disengage attention from the frequent stimulus in the previous side in sessions 3 and 4. Consistent with the idea of Fischer and Breitmeyer (1987), lick latencies were delayed in sessions 3 and 4 requiring attentional disengagement in the present study.

However, the delayed lick latencies in sessions 3 and 4 could be explained by ambiguousness of reward availability due to the distractor effect instead of attentional disengagement. This possibility seems to be not likely based on the following results. First, there was no difference in correct ratios among the five sessions. This indicates that the animals performed similarly sessions 3 and 4 with simultaneous presentation of the infrequent and frequent stimuli as well as sessions 1 and 2 with the infrequent stimulus only, suggesting that ambiguousness was not evident in sessions 3 and 4. The only difference was lick latencies. A human psychological study reported that presentation of a stimulus in a specific position induces attention to that location (cueing effect; Wright and Richard, 2000). The delayed lick latencies in sessions 3 and 4 might be attributed to the cueing effect to the frequent stimulus. Second, the neuronal data also suggest that delayed lick latencies in sessions 3 and 4 might not be ascribed to the simple distractor effect. The distractor effect can be explained by divided attention (e.g., Corbetta et al., 1991). That is, previous studies reported that the neuronal responses to target stimuli with distractors were smaller than those to target stimuli without distractors not only in the SC but also in the parietal and prefrontal cortices (McPeek and Keller, 2002; Suzuki and Gottlieb, 2013). However, response magnitudes of the attention-related neuron in **Figure 6** to the single stimulus (left light) in R1 and the double stimuli (left and right lights) in R3 were similar, suggesting that the divided attention effect was not evident in R3. However, further studies are required to confirm this idea.

### VISUALLY RESPONSIVE SC NEURONS

The SC is a laminated structure, classically divided into superficial, intermediate, and deep layers. The superficial layers receive inputs directly from the retina and V1 (primary visual cortex), and project directly to the deeper layers of the SC and the pulvinar (Hilbig et al., 2000; Doubell et al., 2003; May, 2006). Consistent with the simple connections of the superficial layers, the visually responsive neurons, which were located mainly in the superficial layers, responded to the contralateral light with shorter latencies (26.5 ms) than the other neuronal types. Previous studies reported that the mean response latency to visual stimuli was 21.4 ms in the superficial layers of the SC in Long Evans rats (Fortin et al., 1999), and 50 ms in albino rats (Thomas et al., 2005), which are comparable to our present findings. These neurons might transfer visual information to other types of SC neurons.

### REWARD AND ATTENTION SHIFT-RELATED NEURONS

The reward and attention shift-related neurons, which responded to the contralateral CSs in the reward trials, were located mainly in both the intermediate and deep layers of the SC. The intermediate and deep layers of the SC have intimate connections with various cortical and subcortical structures and relatively few connections with the retina (Tardif and Clarke, 2002; May, 2006). One of the important functions of the SC is attention shifting (Goldberg and Wurtz, 1972; Gattass and Desimone, 1996). The “build-up” or “visuomotor” cells, which are recorded from the intermediate and deep layers of the monkey SC and involved in motor (saccade) preparation (Sparks and Hartwich-Young, 1989), are also involved in attention shifting (Kustov and Robinson, 1996; Ignashchenkova et al., 2004). The locations of the reward and attention shift-related neurons in the present study correspond to the locations where electrical stimulation induces orienting behavior in rats (Sahibzada et al., 1986). The present results suggest that the reward and attention shift-related neurons in the rat SC might correspond to “build-up” or “visuomotor” cells in monkeys, and are involved in attention shifting.

Although the mean response latency of these neurons was longer than that of the visually responsive neurons, some of these neurons had short latencies comparable to visually responsive neurons, which are shorter than cortical neurons (Wang et al., 2014). These neurons might be involved in visual attention independent of the cortex. Consistently, SC inactivation induces behavioral impairments in a covert attentional task through mechanisms that are independent of the classic effects in the visual cortex (Zénon and Krauzlis, 2012). However, being part of the network of brain areas involved in spatial attention, the SC is also a node in descending pathways to guide behaviors to a target (Gandhi and Katnani, 2011; Borra et al., 2014). SC neurons with longer latencies might be controlled by these cortical outputs. These results are consistent with a previous study that found activity of monkey SC neurons in the intermediate and deep layers to be associated with both bottom–up and top–down shifts of attention (Bell and Munoz, 2008). Taken together, through the different (bottom–up and/or top–down) mechanisms, the rodent SC also guides behaviors to attended stimuli, and might output signals through its connections with the dopaminergic system (Redgrave and Gurney, 2006) and the predorsal bundle (Sahibzada et al., 1986).

### ATTENTION DISENGAGEMENT-RELATED NEURONS

The attention disengagement-related neurons responded more strongly to the CSs requiring attention disengagement in sessions 3 and 4 than to other rewarding CSs in sessions 1 and 2, requiring no attention disengagement, although behavioral requirement was the same. The only difference between the sessions 1 and 2 vs. sessions 3 and 4 was the lights; only one of the two lights was turned on in sessions 1 and 2 while two lights were simultaneously turned on in sessions 3 and 4. Therefore, differences in response magnitudes between sessions 1 and 2 vs. sessions 3 and 4 might be ascribed to differences in total luminance of the stimuli. However, this is unlikely since response magnitudes to the two lights in sessions 3 and 4 were significantly larger than response to the same stimuli in session 5, in which attentional disengagement was not required. Furthermore, we discovered that response latencies of attention disengagement-related neurons were faster and response magnitudes stronger specifically in the sessions 3 and 4 requiring attentional disengagement when compared to those in sessions 1, 2, and 5, in which lick latencies were faster than in sessions 3 and 4. These results strongly suggest that activity of attention disengagement-related neurons does not reflect simple motor-preparatory activity. In addition, activity of some attention disengagement-related neurons was negatively correlated with lick latencies in sessions requiring attention disengagement. Together, these results suggest that attention disengagement-related neurons specifically play a role in attentional disengagement processes to guide licking. These characteristics are contrasted with those of the reward and attention shift-related neurons that responded to the contralateral rewarding CSs requiring no attention disengagement.

Another possibility is that the present results could be ascribed to a distractor effect since the animals must detect a target stimulus from the two stimuli in R3 and R4. However, it is not likely based on the following reasons. First, previous studies reported that the neuronal responses to target stimuli with distractors were smaller than those to target stimuli without distractors (see the above section). Therefore, it is noted that the present results in R3 and R4 were opposite to those to the target stimuli with distractors in the previous studies; the neuronal responses of attention disengagement-related neurons to the infrequent stimuli in R3 and R4 were rather larger than those to the infrequent stimuli in R1 and R2. Second, behavioral latencies were negatively correlated with response magnitudes of these attention disengagement-related neurons in R3 and R4. These results suggest that the distractor argument is less likely.

A previous human case study reported that a patient with lesions including the right SC showed deficits in saccades to the contralateral (left) target in an overlap condition requiring disengagement (Pierrot-Deseilligny et al., 1991). These human behavioral data are consistent with the present results in which activity of the attention disengagement-related neurons was associated with disengagement of attention from the ipsilateral light and attentional shift to the contralateral light. Although no previous neurophysiological studies have reported neurons associated with attentional disengagement, the frontal eye fields and parietal lobe, which are implicated in attentional disengagement by behavioral and EEG data in humans (Posner et al., 1984; Rivaud et al., 1994; Csibra et al., 1997), send projections to the deep layer of the SC (Sparks and Hartwich-Young, 1989), the location where the attention disengagement-related neurons were found in the present study. Further studies are required to investigate whether activity of attention disengagement-related SC neurons reflects cortical activity.

Several studies suggest deficits in disengagement of visual attention as a unique feature of autism in young children (Rodier, 2000; Landry and Bryson, 2004; Elsabbagh et al., 2009, 2013). These studies investigated orienting reactions of young autistic and non-autistic children who looked at 3 computer monitors in front of them. Once attention was first engaged on a fixation stimulus in the central monitor, a second stimulus was presented on either side, either simultaneously (overlap condition) or successively (gap condition). Reaction time to the peripheral stimuli was longer in autistic children in the overlap condition compared with non-autistic children. This experimental situation is comparable to the present study, suggesting that the deep layers of SC might be involved in the pathology of autism. Furthermore, some pathological changes in the SC were observed in human autistic patients and animal models of autism (Dendrinos et al., 2011; Kleinhans et al., 2011; Zhao et al., 2013).

### COMPARISON WITH THE PRIMATE SC

In monkey studies, when animals were required to saccade to an eccentric saccadic target from an initially fixated target, introduction of a temporal gap between the disappearance of the initially fixated target and the appearance of the eccentric saccadic target reduces saccadic reaction time (i.e., *gap effect*; Paré and Munoz, 1996; Dorris et al., 1997). The gap effect is well explained by an interaction between fixation-related neuronal activity in the rostral SC and saccade-related neuronal activity (Dorris et al., 1997; Krauzlis et al., 2004); the disappearance of the initially fixated target decreases fixation-related neuronal activity, which facilitates saccade-related neuronal activity. However, this model in the monkey SC might not be directly applicable to the present study. First, the model well explains the gap effect induced by disappearance of a previous fixation target, while the present study investigated mechanisms by which the SC contributes to attention shift during the presence of a previous stimulus (see Introduction). Second, in the present study, rodents were used. Previous studies reported fundamental differences in the visual system between rodents and primates. The rodent SC receives information from the entire retina in the contralateral eye, while the SC in primates receives information from the hemi-retina of both eyes, which are devoted to the contralateral visual field (Heesy, 2009; Kaas, 2014). That is, the primate visual system is more specialized to binocular and central vision, compared with rats. Furthermore, eye movements in freely moving rats are typically non-conjugate to maintain an overhead continuous binocular field rather than target fixation (Wallace et al., 2013). These findings suggest that attentional disengagement mechanisms might not be identical between primates and rodents. Further studies are required to investigate the differences in attentional disengagement mechanisms between rodents and primates.

## CONCLUSION

The present study demonstrated the existence of rodent SC neurons that are comparable to those in monkeys (visually responsive neurons, and reward and attention shift-related neurons), as well as a new type of SC neuron that has not been reported in previous studies (attention disengagement-related neurons). The visual attention system can remain either engaged or disengaged (Fischer and Breitmeyer, 1987) and based on our current findings, we suggest the following neural mechanisms of attentional engagement and disengagement. Reward and attention shift-related neurons might be involved in the engagement process, while attention disengagement-related neurons might be involved in the disengagement process. To attend a contralateral target, attention to the ipsilateral target must be initially disengaged (see above). To disengage attention from the ipsilateral CSs, the attention disengagement-related neurons in the ipsilateral SC might inhibit reward and attention shift-related neurons in the contralateral SC, which are involved in engagement of attention to the ipsilateral target, through the inhibitory tecto-tectal pathway (Goodale, 1973; Munoz and Istvan, 1998), colliculo-thalamo-basal ganglia-collicular loop (McHaffie et al., 2005), or colliculo-basal ganglia-collicular loop (Redgrave and Gurney, 2006). Interestingly, activity of the attention disengagement-related neurons seemed to be inhibited during presentation of the ipsilateral CSs in sessions 1 and 2 requiring no disengagement; response latencies were slower and response durations were shorter in these trials (for left SC: R1 trials in **Figure 4Ab,c**; for right SC: R2 trials in **Figure 4Bb,c**). These results can be interpreted as follows. When an ipsilateral target without disengagement is presented, contralateral reward and attention shift-related neurons are activated to engage attention to the ipsilateral target, and at the same time activity of the attention-disengagement neurons in the ipsilateral SC is suppressed so that these attention disengagement-related neurons do not inhibit the reward and attention shift-related neurons in the contralateral SC. Further studies are required to prove or disprove this idea.

On the other hand, in the present study we required a licking response from the rats and not a saccade. Previous studies reported SC involvement not only in eye movements (saccades), but also in hand control (Borra et al., 2014) and locomotor decisions (Felsen and Mainen, 2008). The present study provides additional evidence with respect to a role of the SC in motor control; the rodent SC is involved in guiding lick behaviors, especially in an attentional disengagement condition.

## Conflict of Interest Statement

The authors declare that the research was conducted in the absence of any commercial or financial relationships that could be construed as a potential conflict of interest.
